# Reporting quality of scoping reviews in dental public health

**DOI:** 10.1186/s12874-023-01863-2

**Published:** 2023-02-27

**Authors:** Lara Dotto, Mateus Bertolini Fernandes dos Santos, Rafael Sarkis-Onofre

**Affiliations:** 1 School of Dentistry, Regional Integrated University of Upper Uruguai and Missions (URI), Erechim, RS Brazil; 2 Graduate Program in Dentistry, Pontifical Catholic University of Rio Grande Do Sul (PUCRS), Porto Alegre, RS Brazil; 3 Graduate Program in Dentistry, Federal University of Pelotas, Pelotas, RS Brazil; 4 Graduate Program in Dentistry, Atitus Educação, 304, Senador Pinheiro St, Passo Fundo, RS 99070-220 Brazil

**Keywords:** Public health, Dentistry, Scoping review, Methods, Reporting

## Abstract

**Background:**

The study aimed to explore reporting characteristics of scoping reviews in dental public health and the impact of some factors on the reporting quality.

**Methods:**

This study searched for dental public health scoping reviews in PubMed and Scopus without year restrictions and restricted to English-language publications. Study selection was undertaken by two reviewers independently. One reviewer, after training, extracted data from included studies considering general study characteristics and reporting characteristics. The impact of PRISMA-ScR publication, journal endorsement, and use of study protocol on the reporting was explored.

**Results:**

Eighty-one scoping reviews were included. Five items presented rates of appropriate reporting higher than 80% considering the overall percentage. Related to the impact of PRISMA-ScR publication, six items were found more often in scoping reviews published after the publication of PRISMA-ScR than in scoping reviews published before the publication of PRISMA-ScR. With regards to journals endorsement, only two reporting characteristics were found more often in scoping reviews published in journals that endorse the PRISMA-ScR statement than in scoping reviews published in non-endorsers journals. Last, regarding the use of the pre-specified protocol, five reporting characteristics presented differences in studies reporting the use of pre-specified protocol than in studies that did not mention the use of a protocol. All differences were statistically significant.

**Conclusions:**

Important information is missing in the included scoping reviews demonstrating crucial reporting problems.

**Supplementary Information:**

The online version contains supplementary material available at 10.1186/s12874-023-01863-2.

## Background

In an ever-growing science field, with a lot of scientific evidence being published throughout different platforms and journals daily [1], the gathering of adequate scientific evidence should be made to help the decision-making process for public health policies and at the individual level. However, the increased number of publications or the lack of scientific criteria of some publications might impair a sound decision process. In this way, it is important to highlight that evidence-based public health policies present several benefits, such as better treatment results, greater workforce productivity, and more efficient use of resources [2], and that health professionals that comply with the best available evidence tend to obtain better results in their practice [3–5].

The scoping reviews are an important study methodology that can help the evidence-based public health policies. They were developed as a tool to gather and synthesize scientific evidence regarding a certain topic, being specifically indicated to map the best available scientific evidence, identify knowledge gaps, or investigate research conduct [6, 7]. Although scoping reviews have become a popular approach for evidence gathering and synthetization, previous studies have pointed out the need for scoping reviews methodological standardization to ensure the utility and strength of the synthesized evidence [6, 8].

Previous studies have reported that a considerable number of studies is often poorly reported [9, 10], which might result in biased or invalidated conclusions leading to loss of time and resources. Considering that, in 2018, the Preferred Reporting Items for Systematic Reviews and Meta-Analyses (PRISMA) extension for scoping reviews has published, containing 20 essential reporting items to include when completing a scoping review to increase study transparency and ensure that the results are trustworthy [11].

In dental public health, there are no studies assessing the reporting quality of scoping reviews. Thus, the study's objective was to explore reporting characteristics of scoping reviews in dental public health by assessing the items recommended by the PRISMA Extension for Scoping Reviews (PRISMA-ScR) [11] and the influence of some factors on the reporting quality.

## Methods

The study protocol was registered on the Open Science Framework platform and is available at the following link: https://osf.io/6vykb. In the initial analysis of this project [12], we assessed the reporting of authors’ justifications for choosing the scoping review methodology considering all dental specialties, and our findings demonstrated that most scoping reviews did not report the rationale for choosing that method. Also, we identified that Dental Public Health had the most publications among the dental specialties and decided to update the search and explore other reporting characteristics of scoping reviews in that dental specialty.

### Eligibility criteria

We considered a scoping review, articles mentioning in the title, abstract, introduction or methodology that a scoping review/search/study/exercise was conducted or studies conducting mapping reviews or literature mapping. Studies were included regardless of the year of publication and methodology or reporting quality.

We considered “Dental Public Health” as the science and art of preventing and controlling dental diseases and promoting dental health through organized community efforts [13]. Articles reporting narrative reviews, systematic reviews, assessing study quality, overviews, commentaries, and scoping review protocols were excluded.

### Search strategy

Table 1 presents the search strategy used. We conducted searches in PubMed and Scopus, restricted to English results. The authors created the search strategy based on the Mesh terms of PubMed e adapted it to the other database. The last search was conducted on March 15, 2022.

**Table 1:** Search strategy

PubMed
"Oral Health"[Mesh] OR "Oral Health" OR "Health, Oral" OR "Dentistry"[Mesh] OR "Dentistry" OR "Dental Research"[Mesh] OR "Dental Research" AND “scoping review” OR “scoping reviews” OR “scoping search” OR “scoping search” OR “scoping study” OR “scoping studies” OR “scoping exercise” OR “mapping review” OR “mapping reviews” OR “mapped review” OR “mapped reviews” OR “literature mapping”
Scopus
"Oral Health" OR "Health, Oral" OR "Dentistry" OR "Dental Research" AND “scoping review” OR “scoping reviews” OR “scoping search” OR “scoping search” OR “scoping study” OR “scoping studies” OR “scoping exercise” OR “mapping review” OR “mapping reviews” OR “mapped review” OR “mapped reviews” OR “literature mapping”

### Screening

Results of searches were transferred to Endnote software, where duplicate studies were removed. After, the registers were uploaded to the DistillerSR, an online software that automates all study stages. Using that software, two researchers independently identified the article by assessing the titles and abstracts for relevance. Articles were classified as “included”, “excluded”, and “insufficient information”. Records classified as “included” and “insufficient information” were selected to assess the full texts. Next, the same two reviewers assessed the full texts independently based on the eligibility criteria. Discrepancies in the titles, abstract assessment, and full-text analysis were solved by consensus.

### Data collection

A standardized data extraction form was created in the DistillerSR. All data mentioned in the previous protocol were collected. In addition, we collected 20 reporting items recommended by the PRISMA-ScR^11^ (Supplemental Material). Each checklist item was classified as “fully reported”, “partially complete,” or “not reported”. The two items related to the critical appraisal of individual sources of evidence (Methods and Results) were not assessed because, based on the JBI Manual for Evidence Synthesis, it is not a mandatory step. Regarding the journal endorsement of the PRISMA-ScR, we verified the information on the PRISMA website (http://www.prisma-statement.org/Endorsement/PRISMAEndorsers#d) and on the journals’ instructions to authors. In the journals’ instructions to authors, we considered a journal endorsement any mention of the PRISMA statement or EQUATOR Network. Initially, a pilot test was conducted between two reviewers to ensure consistency in data extraction. After, one reviewer extracted all data, and another verified it.

### Data analysis

First, descriptive statistics were performed to present the data of characteristics of included studies. The PRISMA-ScR was published in September 2018, and we categorized the included studies as studies published before September 2018, studies published between September 2018 and September 2019, and after September 2019. We decided to use this categorization to consider one year (between September 2018 and September 2019) as the time of adoption of the PRISMA-ScR by authors and journals.

Second, we calculated the percentage of studies with adequate reporting for each item considering the three previously mentioned categories and if the item was classified as “fully reported”, “partially complete,” or “not reported”.

Last, we explored differences in the reporting of all items before and after the PRISMA-ScR publication, the reporting between studies published in journals endorsing the PRISMA-ScR and journals not endorsing the PRISMA-ScR and compared studies reporting the protocol register and studies that did not report the protocol register. For these analyses, each PRISMA-ScR item will be categorized as ‘‘reported’’ or ‘‘not reported.’’ In situations where the item was judged as ‘unclear’ reported, it will not be considered in the analysis, and if an item was partially complete, it will be classified as “not reported”. Thus, the proportion of scoping reviews with adequate reporting of each item was calculated. Using this proportion, we compared the reporting between studies published before September 2018 and those published after September 2019, the reporting between studies published in journals endorsing the PRISMA-ScR and journals not endorsing the PRISMA-ScR and compare studies reporting the protocol register and studies that did not report the protocol register with the calculation of the (RR) Risk Ratio of a 95% confidence interval for each characteristic. An RR greater than 1 will indicate increased reporting of the item. The analysis will be performed using Review Manager Web (The Cochrane Collaboration, 2021).

## Results

**Fig. 1:**
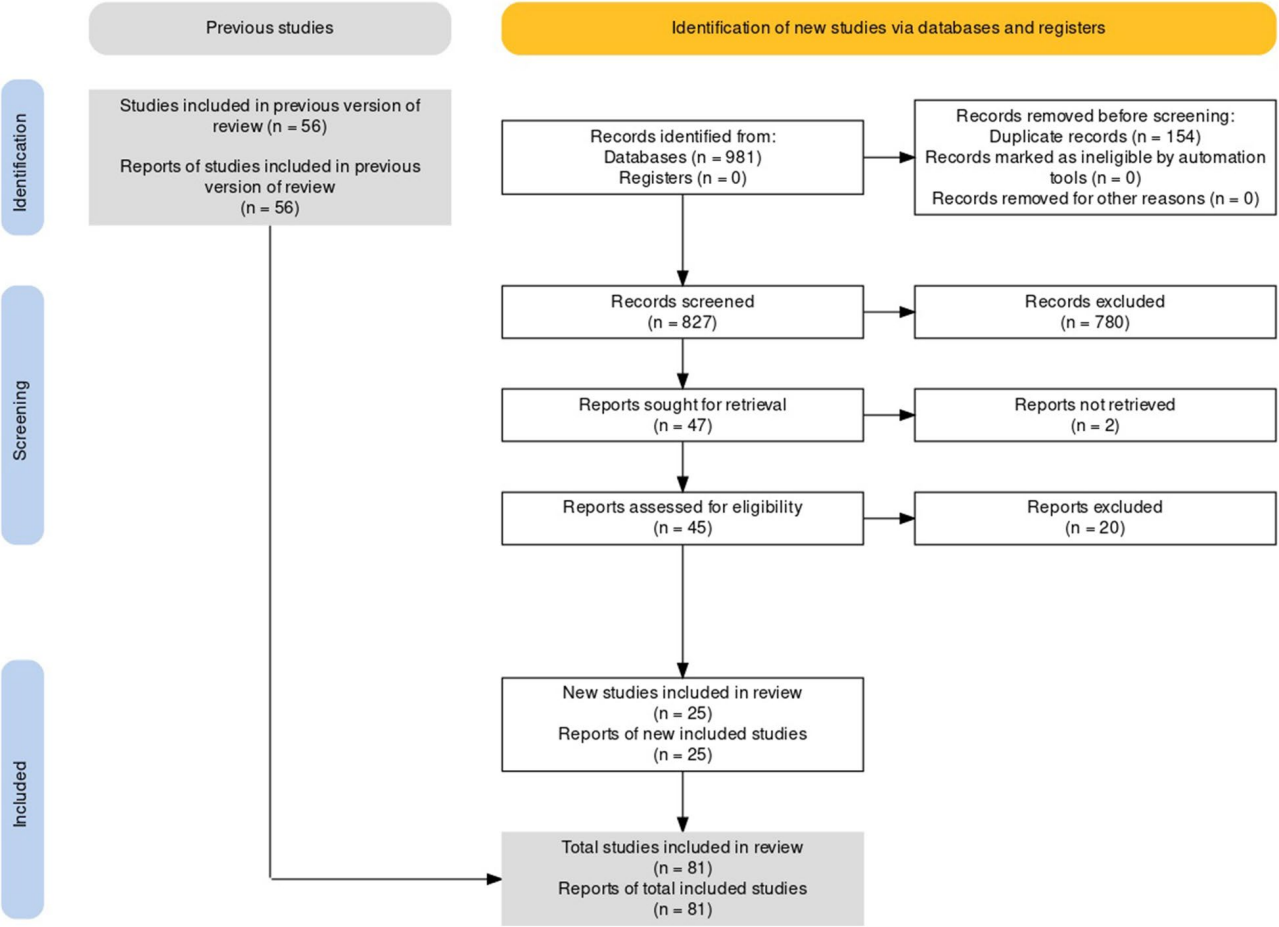
Flow diagram of study selection

Initially, 56 reports were obtained from the previous search. After updating the search, 25 reports were identified yielding 81 studies that fulfilled the inclusion criteria. Figure 1 presents the flow diagram of study selection, and the Supplemental Material presents the list of excluded studies.

Table 2 presents the characteristics of the included studies. Australia, United Kingdom, the United States of America (USA), and Canada were the countries with the most contributions, representing 69.1% of the sample. The articles were published in 47 journals with Community Dental Health (*n* = 8, 9.9%) and BMC Oral Health (*n* = 7, 8.6%) being the journals with the most published scoping reviews. Forty-nine articles (60.5%) were published in journals that endorse the PRISMA-ScR statement, thirty-one articles (38.3%) reported a non-profit sponsor, and 29 (35.8%) did not report funding details. The methodological guide that was most used was the Arksey and O’Malley, cited in 43 studies (53.1%). Considering only studies published after the PRISMA-ScR publication, most of them did not mention the use of such reporting guideline (*n* = 32, 60.4%).

**Table 2:** Characteristics of included studies

**Country of corresponding author (*n* = 81)**	***n***	**%**
Australia	19	23.5%
United Kingdom	14	17.3%
United States of America	13	16%
Canada	10	12.3%
Brazil	6	7.4
Iran	6	7.4
Chile	2	2.5
Germany	1	1.2
Indonesia	1	1.2
Ireland	1	1.2
Italy	1	1.2
Japan	1	1.2
Netherlands	1	1.2
Nigeria	1	1.2
Saudi Arabia	1	1.2
Singapore	1	1.2
South Africa	1	1.2
Taiwan	1	1.2
**Journal (*n* = 81)**		
Community Dental Health	8	9.9%
BMC Oral Health	7	8.6%
Journal of Dentistry	4	4.9%
Journal of Public Health Dentistry	4	4.9%
Gerodontology	3	3.7%
International Journal of Environmental Research and Public Health	3	3.7%
Special Care in Dentistry	2	2.5%
Australian Dental Journal	2	2.5%
BMC Public Health	2	2.5%
British Dental Journal	2	2.5%
Community Dentistry and Oral Epidemiology	2	2.5%
International Journal of Dental Hygiene	2	2.5%
Journal of Dental Education	2	2.5%
Journal of the Canadian Dental Association	2	2.5%
Plos One	2	2.5%
Systematic Reviews	2	2.5%
Journal of the American Dental Association	2	2.5%
Annali di Stomatologia	1	1.2%
Annals of Ibadan Postgraduate Medicine	1	1.2%
BMC Health Services Research	1	1.2%
BMJ Open	1	1.2%
Brazilian Oral Research	1	1.2%
Cost Effectiveness and Resource Allocation	1	1.2%
Dentistry Journal	1	1.2%
Diabetes Research and Clinical Practice	1	1.2%
European Journal of Oral Sciences	1	1.2%
Globalization and Health	1	1.2%
Health Services Insights	1	1.2%
International Journal for Equity in Health	1	1.2%
International Journal of Clinical Pediatric Dentistry	1	1.2%
International Journal of Paediatric Dentistry	1	1.2%
Journal of Clinical Nursing	1	1.2%
Journal of Oral Rehabilitation	1	1.2%
Journal of Patient Safety	1	1.2%
Journal of Prosthodontic Research	1	1.2%
Medical Care Research and Review	1	1.2%
Obesity Research & Clinical Practice	1	1.2%
Oral Diseases	1	1.2%
Oral Health and Preventive Dentistry	1	1.2%
Orthodontics & Craniofacial Research	1	1.2%
Population Health	1	1.2%
Quality of Life Research	1	1.2%
Quintessenz International	1	1.2%
Research in Developmental Disabilities	1	1.2%
JDR Clinical & Translational Research	1	1.2%
Trauma, Violence, & Abuse	1	1.2%
Revista Ciência e Saúde Coletiva	1	1.2%
**Articles published in journals endorsing the PRISMA-ScR Statement (*n* = 81)**	49	60.5%
**Terms mentioned in the title or abstract#**	***n***	**%**
Scoping review	76	93.8%
Scoping search	1	1.2%
Scoping study	3	3.7%
Mapping review	2	2.5%
Neither	1	1.2%
**Funding**	**n**	**%**
Non-profit sponsor	31	38.3%
Not reported	29	35.8%
No funding	18	22.2%
Mixed	2	2.5%
Unclear	1	1.2%
**Methodology guide mentioned# (*n* = 81)**	**n**	**%**
Arksey and O’Malley	43	53.1
Levac	12	14.8
JBI	15	18.5
Other	21	25.9
PRISMA-SCR	9	
PRISMA	5	
Moher et al	1	
PRISMA and the published protocol	1	
PRISMA flow diagram	1	
Peters et al	3	
Sucharew et al	1	
Not reported	14	17.3
**Use of PRISMA-SCR (considering articles published since 2019 January *n* = 53)**	***n***	**%**
No	32	60.4%
Yes	21	39.6%
**How PRISMA was used (considering the 22 studies that mentioned used the PRISMA-ScR *n* = 22)**	***n***	**%**
Methodology guideline	5	23.8%
Reporting guideline	7	33.3%
Neither	9	42.9%
#Could be selected more than one answer		

**Fig. 2:**
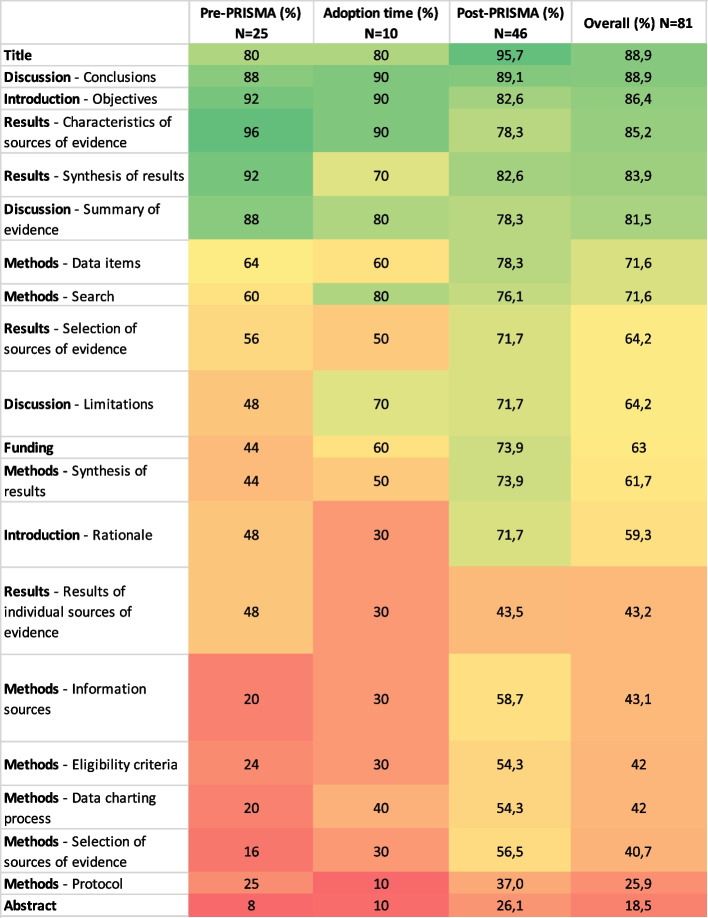
The percentage of adequately reported items before and after the PRISMA-ScR statement publication and the overall rate. Each cell is colored according to the reporting using the transformation of three colors: red (0%), yellow (50%), and green (100%)

The percentage of adequately reported items before and after the PRISMA-ScR statement publication and the overall rate are presented in Fig. 2. Considering the overall percentage, items such as title, conclusions, objectives, characteristics of sources of evidence, synthesis of results (results section), and summary of evidence presented rates of adequately reporting higher than 80%.

**Fig. 3:**
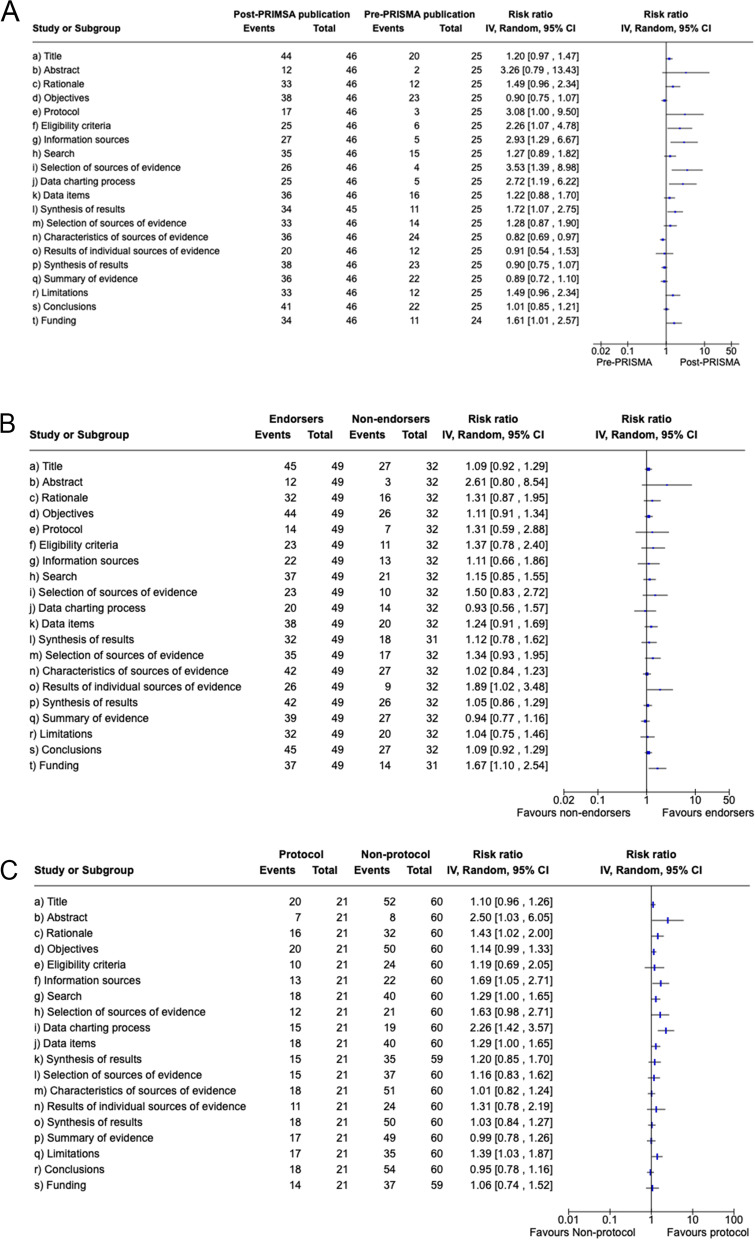
Results related to the reporting of items before and after the PRISMA-ScR publication (**a**), the reporting between studies published in journals endorsing the PRISMA-ScR and journals not endorsing the PRISMA-ScR (**b**) and compared studies reporting the protocol register and studies that did not report the protocol register (**c**)

Figure 3 presents the results related to the reporting of items before and after the PRISMA-ScR publication (a), the reporting between studies published in journals endorsing the PRISMA-ScR and journals not endorsing the PRISMA-ScR (b) and compared studies reporting the protocol register and studies that did not report the protocol register (c). Related to the impact of PRISMA-ScR publication, six items were better described in scoping reviews after the publication of PRISMA-ScR (eligibility criteria, information sources, selection of sources of evidence, data charting process, synthesis of results in the methods section, and funding) than scoping reviews published before the publication of PRISMA-ScR. With regards to the journal endorsement, only two reporting characteristics were improved in scoping reviews published in journals that endorse the use of PRISMA-ScR statement (results of individual sources of evidence and funding) than in scoping reviews published in non-endorser journals. Last, regarding to the use of the pre-specified protocol, five reporting characteristics were better described in studies reporting the use of pre-specified protocol (abstract, rationale, information sources, data charting process, and limitations) than in studies that did not mention the use of a protocol. The risk relative and 95% confidence interval of all analysis are presented in the Fig. 3.

## Discussion

To the best of our knowledge, this study is the first in oral health literature to explore the reporting characteristics of scoping reviews in dental public health and factors that could influence the reporting quality. Our results indicated that crucial aspects are missing in the included studies demonstrating a potential for improvement. Also, our findings revealed a slight influence of the PRISMA-ScR publication, journal endorsement of the PRISMA-ScR, and study protocol development on the reporting quality.

Our results demonstrated that six reporting items from the PRISMA-ScR presented rates of adequate reporting higher than 80%; however, most of the crucial aspects of methods presented rates smaller than 50%, which could impair a proper understanding by readers and the replicability by researchers. There are some important explanations for this result. First, it could be associated with a word count restriction imposed by some journals. Second, some essential information could be reported in the study protocol, and third, could be related to researchers to be unaware of the scoping review methodology.

Other results of reporting items that should be mentioned are related to the sources of funding and the abstract. The reporting of sources of funding presented an overall rate of 63%, which may be associated with the journal policies that request authors to indicate funding details in the submission platform or in the cover letter; however, these details are not published in the main article. The results of abstract reporting were the worst. Although PRISMA-ScR recommends seven essential items to be considered in the abstract [11], many journals impose word count restrictions varying from 200 to 350 words jeopardizing such reporting.

Aspects related to the PRISMA-ScR publication and the endorsement by journals presented a small influence on our results and this finding is aligned with a previous study by Veroniki et al. (2021) [14]. There are some critical explanations for these results. First, we can observe an increase in the number of journals endorsing reporting guidelines; however, some journals only mention that authors should use the reporting guidelines available in the EQUATOR Network library or do not specify the use of the PRISMA-ScR for scoping reviews. Second, the authors reported the use of PRISMA-ScR based on previous publications or journal requirements without adequate knowledge of its use and, in these cases, we can expect the “passive act of filling up the PRISMA-ScR checklist”.

The importance of study protocol and its impact on the reporting quality was already highlighted in previous publications [14, 15]. However, our findings demonstrated a small effect of the study protocol on the reporting quality of scoping reviews, this result could be attributed to a lack of knowledge of researchers on how to develop a protocol and what is the role of a study protocol. Also, some studies reported the protocol development but mentioned that it was not publicly available, hindering the access and assessment of such information.

Our study presents some limitations that should be mentioned. First, we did not assess the protocols for relevant information; however, we considered the supplementary materials and appendices of all included studies. Second, the journal's word count restrictions were not assessed and considered in the analysis. Third, items classified as partially reported were considered as not reported in the analysis; however, we observed many items with a low rate of adequate reporting, which could demonstrate a minor influence of this classification on the results. Last, the temporal differences observed cannot be attributed with certainty to the publication of PRISMA-ScR, as well as the association between journal endorsement and adherence to the reporting guideline since this is an observational study.

Finally, the reporting of scoping reviews should be improved, and we believe that the first step is a better understanding of the role of scoping reviews in health research, especially in the oral health area, which was studied in this paper. Also, the use of PRISMA-ScR is an important step toward improving the reporting quality and should be encouraged by journals through a clear statement and a mandatory recommendation in the instructions to authors and by researchers, students, editors, and publishers through educational initiatives. However, it is important to mention that if the scoping reviews are poorly designed and conducted, it does not matter how well-reported the results and discussion are. Thus, it is essential that study design, conduct, and reporting be aligned to improve study reproducibility and reliability.

In general, important information is missing in the included studies demonstrating crucial reporting problems. We could not observe a significant impact of the PRISMA-ScR publication, journal endorsement of the PRISMA-ScR, and the development of study protocol in the reporting quality, indicating that better dissemination of these tools is necessary.

## Supplementary Information

**Additional file 1.**

## Data Availability

All data generated or analyzed during this study are included in this published article.

## Abbreviations

PRISMA: Preferred Reporting Items for Systematic Reviews and Meta-Analyses
PRISMA-ScR: PRISMA Extension for Scoping Reviews
RR: Risk ratio
USA: United States of America
