# Supplementary material for: Reporting quality of scoping reviews in dental public health
Source: BMC Med Res Methodol. 2023 Feb 27;23:53. doi: 10.1186/s12874-023-01863-2 (PMC9972695; doi:10.1186/s12874-023-01863-2)
Supplement: Supplementary file 1 — Additional file 1. [file 12874_2023_1863_MOESM1_ESM.docx]

**Supplemental material**

| **PRISMA-ScR items** | |
| --- | --- |
| **PRISMA-ScR domains** | **Score as “reported”** |
| 1. Title | If scoping review, mapping review, or similar are reported. |
| 1. Structured summary (abstract) | If there is a structured summary, regardless of the items included in the summary. |
| 1. Rationale | If described the rationale for the review in the context of what is already known. Explain why the review questions/objectives lend themselves to a scoping review approach. |
| 1. Objectives | If provided an explicit statement of the questions and objectives being addressed with reference to their  key elements or other relevant key  elements used to conceptualize the review questions and/or objectives. |
| 1. Protocol | If the existence of protocol is reported |
| 1. Eligibility criteria | If specified characteristics of the sources of evidence used as eligibility criteria (e.g., years considered,  language, and publication status), and provide a rationale. |
| 1. Information sources | If described all information sources in the search as well as the date the most recent search was executed. |
| 1. Search | If presented the full electronic search strategy for at least 1 database. |
| 1. Selection of sources of evidence | If reported the screening process (methods). |
| 1. Data charting process | If described the methods of charting data from the included sources of evidence and any processes for obtaining and confirming data from investigators (methods). |
| 1. Data items | If reported a list and define all variables for which data were sought and any assumptions and simplifications made. |
| 1. Synthesis of results | If described the methods of handling and summarizing the data that were charted |
| 1. Selection of sources of evidence | If the number of sources of evidence screened, assessed for eligibility, and included in the review, with reasons for exclusions at each stage, ideally using a flow diagram were reported. |
| 1. Characteristics of sources of evidence | If for each source of evidence, presented characteristics for which data were charted and provided the citations. |
| 1. Results of individual sources of evidence | For each included source of evidence, presented the relevant data that were charted that relate to the  review questions and objectives. |
| 1. Synthesis of results | If summarized and/or presented the charting results as they relate to the review questions and objectives. |
| 1. Summary of evidence | If summarized the main results, linked to the review questions and objectives, and considered the relevance to key groups. |
| 1. Limitations | If discussed the limitations of the scoping review process. |
| 1. Conclusions | If provided a general interpretation of the results with respect to the review questions and objectives, as well as potential implications and/or next steps |
| 1. Funding | If described sources of funding and the role of funders. |

**List of included studies**

1. Ghanbarzadegan A, Bastani P, Luzzi L, Brennan D. Inequalities in utilization and provision of dental services: a scoping review. *Syst Rev* 2021;10(1):222.

2. Martin N, Sheppard M, Gorasia GP, Arora P, Cooper M, Mulligan S. Drivers, opportunities and best practice for sustainability in dentistry: A scoping review. *J Dent* 2021;112:103737.

3. Cenzato N, Nobili A, Maspero C. Prevalence of Dental Malocclusions in Different Geographical Areas: Scoping Review. *Dent J (Basel)* 2021;9:117.

4. Badewy R,Singh H,Quiñonez C,Singhal S. Impact of Poor Oral Health on Community-Dwelling Seniors: A Scoping Review. *Health Serv Insights* 2021;14:1178632921989734.

5. Hu J, McMillan SS, El-Den S, O'Reilly CL, Collins JC, Wheeler AJ. A scoping review of pharmacy participation in dental and oral health care. *Community Dent Oral Epidemiol* 2021;23.

6. Tan SHX, Lee CKJ, Yong CW, Ding YY. *Gerodontology* 2021;38:351-365. Scoping review: Facilitators and barriers in the adoption of teledentistry among older adults.

7. Martin N, Sheppard M, Gorasia GP, Arora P, Cooper M, Mulligan S. Awareness and barriers to sustainability in dentistry: A scoping review. *J Dent*. 2021;112:103735.

8. Zinah E, Al-Ibrahim HM. Oral health problems facing refugees in Europe: a scoping review. *BMC Public Health* 2021;21:1207.

9. Pitchon A, Gomes VE, Ferreira EFE. Salutogenesis in oral health research in preschool children: A scoping review. *Int J Paediatr Dent* 2021;31:372-382.

10. Wang MC, Wu JY, Shih WY. A scoping review of intervention components of school-based oral health-related behavioural interventions using the Theoretical Domains Framework. *Eur J Oral Sci* 2022;130:e12841.

11. Lansdown K, Irving M, Coulton KM, Smithers-Sheedy H. A scoping review of oral health outcomes for people with cerebral palsy. *Spec Care Dentist* 2022;42:232-243.

12. Núñez MDRR, Raulino ML, Castro RG, de Mello ALSF. Dental plaque control strategies for the elderly population: A scoping review. *Int J Dent Hyg* 2022;20:167-181.

13. Ben-Omran MO, Livinski AA, Kopycka-Kedzierawski DT,Boroumand S,Williams D, Weatherspoon DJ, Iafolla TJ, Fontelo P, Dye BA. The use of teledentistry in facilitating oral health for older adults: A scoping review. *J Am Dent Assoc* 2021;152:998-1011.e17.

14. Oishi MM, Childs CA, Gluch JI, Marchini L. Delivery and financing of oral health care in long-term services and supports: A scoping review. *J Am Dent Assoc* 2021;215-223.e2.

15. Ensaldo-Carrasco E, Suarez-Ortegon MF, Carson-Stevens A, Cresswell K, Bedi R, Sheikh A. Patient Safety Incidents and Adverse Events in Ambulatory Dental Care: A Systematic Scoping Review. *J Patient Saf* 2021;17:381-391.

16. Ramamurthy P, Sharma D, Thomson P. Oral cancer awareness in patients attending university dental clinics: A scoping review of Australian studies. *Aust Dent J* 2022;67:5-11.

17. Bastani P, Mohammadpour M, Ghanbarzadegan A, Rossi-Fedele G, Peres MA. Provision of dental services for vulnerable groups: a scoping review on children with special health care needs. *BMC Health Serv Res* 2021;21:1302.

18. Bastani P, Mohammadpour M, Ghanbarzadegan A, Kapellas K, Do LG. Global concerns of dental and oral health workers during COVID-19 outbreak: a scope study on the concerns and the coping strategies. *Syst Rev* 2021;10(1):45.

19. Sarilita E, Setiawan AS, Mossey PA. Orofacial clefts in low- and middle-income countries: A scoping review of quality and quantity of research based on literature between 2010-2019. *Orthod Craniofac Res* 2021;24:421-429.

20. Shahid M, Shum JH, Tadakamadla SK, Kroon J, Peres MA. Theoretical evidence explaining the relationship between socio-demographic and psychosocial barriers on access to oral health care among adults: A scoping review. *J Dent* 2021;107:103606.

21. Huang SS, Veitz-Keenan A, McGowan R, Niederman R. What is the societal economic cost of poor oral health among older adults in the United States? A scoping review. *Gerodontology* 2021;38:252-258.

22. Kuipers S, Boonstra N, Kronenberg L, Keuning-Plantinga A, Castelein S. Oral Health Interventions in Patients with a Mental Health Disorder: A Scoping Review with Critical Appraisal of the Literature. *Int J Environ Res Public Health* 2021;18(15):8113.

23. Yonenaga K, Itai S, Hoshi K. Implications for clinical dental practice during the coronavirus disease pandemic: A scoping review. *J Prosthodont Res* 2022;66(1):6-11.

24. Garcia DT, Lawson JA, Brody ER, McKernan SC, Raskin SE, Arauz NR, Mosavel M, Brickhouse TH. A scoping review of the roles, training, and impact of community health workers in oral health. *Community Dent Health* 2021;38:198-208.

25. Ghanbarzadegan A, Balasubramanian M, Luzzi L, Brennan D, Bastani P. Inequality in dental services: a scoping review on the role of access toward achieving universal health coverage in oral health. *BMC Oral Health* 2021;21:404.

26. Bastani P, Mohammadpour M, Mehraliain G, Delavari S, Edirippulige S. What makes inequality in the area of dental and oral health in developing countries? A scoping review. *Cost Eff Resour Alloc* 2021;19:54*.*

27. Gupta A, Akiya K, Glickman R, Silver D, Pagán JA. How Patient-Centered Medical Homes Integrate Dental Services Into Primary Care: A Scoping Review. *Med Care Res Rev* 2022;79:487-499.

28. Andrew L, Wallace R, Wickens N, Patel J. Early childhood caries, primary caregiver oral health knowledge and behaviours and associated sociological factors in Australia: a systematic scoping review. *BMC Oral Health* 2021;21:521.

29. Abdelrahman M, Hsu KL, Melo MA, Dhar V, Tinanoff N. Mapping evidence on early childhood caries prevalence: complexity of worldwide data reporting. *Int J Clin Pediatr Dent* 2021;14:1-7.

30. da Silva Sobrinho AR, Ramos LFS, Maciel YL, Maurício HDA, Cartaxo RDO, Ferreira SJ, Sette-de-Souza PH. Orofacial features in children with microcephaly associated with Zika virus: A scoping review. *Oral Dis* 2022;28:1022-1028.

31. Abuhaloob L, Carson S, Richards D, Freeman R. Community-based nutrition intervention to promote oral health and restore healthy body weight in refugee children: A scoping review. *Community Dent Health* 2018;35:81-88.

32. Ajwani S, Jayanti S, Burkolter N, Anderson C, Bhole S, Itaoui R, George A. Integrated oral health care for stroke patients - a scoping review. *J Clin Nurs* 2017;26:891-901.

33. Amarasena N, Haag D, Peres KG. A scoping review of caries risk management protocols in Australia and New Zealand. *Aust Dent J* 2019;64:19-26.

34. Amilani U, Carter HE, Senanayake S, Hettiarachchi RM, McPhail SM, Kularatna S. A scoping review of cost-effectiveness analyses of school-based interventions for caries. *Community Dent Oral Epidemiol* 2020;48:357-363.

35. Beaton L, Humphris G, Rodriguez A, Freeman R. Community-based oral health interventions for people experiencing homelessness: A scoping review. *Community Dent Health* 2020;37:150-160.

36. Bracksley-O'Grady SA, Dickson-Swift VA, Anderson KS, Gussy MG. Health promotion training in dental and oral health degrees: a scoping review. *J Dent Educ* 2015;79:584-91*.*

37. Bradbury-Jones C, Isham L, Morris AJ, Taylor J. The "Neglected" Relationship Between Child Maltreatment and Oral Health? An International Scoping Review of Research. *Trauma Violence Abuse* 2021;22:265-276.

38. Campos V, Cartes-Velasquez R, McKee M. Oral health and dental care in deaf and hard of hearing population: A scoping review. *Oral Health Prev Dent* 2020;18:417-425.

39. Como DH, Stein Duker LI, Polido JC, Cermak SA. The Persistence of Oral Health Disparities for African American Children: A Scoping Review. *Int J Environ Res Public Health* 2019;16:710.

40. Correâ-Faria P, Viana KA, Raggio DP, Hosey MT, Costa LR. Recommended procedures for the management of early childhood caries lesions-A scoping review by the Children Experiencing Dental Anxiety: Collaboration on Research and Education (CEDACORE). *BMC Oral Health* 2020;20:75.

41. Cosgrave C, Malatzky C, Gillespie J. Social Determinants of Rural Health Workforce Retention: A Scoping Review. *Int J Environ Res Public Health* 2019;16:314.

42. Dickson-Swift V, Kenny A, Farmer J, Gussy M, Larkins S. Measuring oral health literacy: A scoping review of existing tools. *BMC Oral Health* 2014;14:148.

43. Dickson-Swift V, Kenny A, Gussy M, de Silva AM, Farmer J, Bracksley-O'Grady S. Supervised toothbrushing programs in primary schools and early childhood settings: A scoping review. *Community Dent Health* 2017;34:208-225.

44. Dooley D, Moultrie NM, Sites E, Crawford PB. Primary care interventions to reduce childhood obesity and sugar-sweetened beverage consumption: Food for thought for oral health professionals. *J Public Health Dent* 2017;77:S104-S127.

45. Doughty J, Lala R, Marshman Z. The dental public health implications of cosmetic dentistry: a scoping review of the literature. *Community Dent health* 2016;33:218-224.

46. Eow J, Duane B, Solaiman A, Hussain U, Lemasney N, Ang R, O’kelly-Lynch N, Girgis G, Collazo L, Johnston B. What evidence do economic evaluations in dental care provide? A scoping review. *Community Dent Health* 2019;36:118-125.

47. Gomez-Rossi J, Hertrampf K, Abraham J, Gaßmann G, Meyer G, Schlattmann P, Göstemeyer G, Schwendicke F. Interventions to improve oral health of older people: A scoping review. *J Dent* 2020;101:103451.

48. Goodell KH, Ticku S, Fazio SB, Riedy CA. Entrustable Professional Activities in Oral Health for Primary Care Providers Based on a Scoping Review. *J Dent Educ* 2019;83:1370-1381.

49. Greenberg BL, Glick M, Tavares M. Addressing obesity in the dental setting: What can be learned from oral health care professionals' efforts to screen for medical conditions. *J Public Health Dent* 2017;77:S67-S78.

50. Gupta A, Keuskamp D. Use and misuse of mixed methods in population oral health research: A scoping review. *Community Dent Health* 2018;35:109-118.

51. Harnagea H, Couturier Y, Shrivastava R, Girard F, Lamothe L, Bedos CP, Emami E. Barriers and facilitators in the integration of oral health into primary care: a scoping review. *BMJ Open* 2017;7:e016078.

52. Harnagea H, Lamothe L, Couturier Y, Esfandiari S, Voyer R, Charbonneau A, Emami E. From theoretical concepts to policies and applied programmes: The landscape of integration of oral health in primary care. *BMC Oral Health* 2018;18(1):23.

53. Jesus RM, Campos FL, Rodrigues LG, Perazzo MF, Soares Ards Ribeiro MTF, Sampaio AA, Ferreira RC. Guideline for oral care of dependent elders: mapping review and cross-cultural adaptation to Portuguese-Brazil. *Braz Oral Res* 2020;34:e097.

54. Kathree BA, Khan SB, Ahmed R, Maart R, Layloo N, Asia-Michaels W. COVID-19 and its impact in the dental setting: A scoping review. *PLoS One* 2020;15:e0244352.

55. Keboa MT, Hiles N, Macdonald ME. The oral health of refugees and asylum seekers: a scoping review. *Global Health* 2016;12:59.

56. Knevel RJM, Gussy MG, Farmer J. Exploratory scoping of the literature on factors that influence oral health workforce planning and management in developing countries. *Int J Dent Hyg* 2017;15:95-105.

57. Kwok C, McIntyre A, Janzen S, Mays R, Teasell R. Oral care post stroke: A scoping review. *J Oral Rehabil* 2015;42:65-74.

58. MacEntee MI, Kazanjian A, Kozak JF, Hornby K, Thorne S, Kettratad-Pruksapong M. A scoping review and research synthesis on financing and regulating oral care in long-term care facilities. *Gerodontology* 2012;29:e41-52.

59. Makansi N, Blaizot A, Vergnes JN, Morris M, Rousseau J, Bedos C. A scoping review on dental clinic accessibility for people using wheelchairs. *Spec Care Dentist* 2021;41:329-339.

60. Mallonee LF, Boyd LD, Stegeman C. A scoping review of skills and tools oral health professionals need to engage children and parents in dietary changes to prevent childhood obesity and consumption of sugar-sweetened beverages . *J Public Health Dent* 2017;77:S128-S135.

61. Mariño R, Zaror C. Economic evaluations in water-fluoridation: a scoping review. *BMC Oral Health* 2020;20:115.

62. Marshall A, Loescher A, Marshman Z. A scoping review of the implications of adult obesity in the delivery and acceptance of dental care. *Br Dent J* 2016;221:251-5.

63. Moore D, Keat R. Does dental appearance impact on employability in adults? A scoping review of quantitative and qualitative evidence. *Br Dent J* 2020 [Online ahead of print.]

64. Naseem M, Shah AH, Khiyani MF, Khurshid Z, Zafar MS, Gulzar S, AlJameel AH, Khalil HS. Access to oral health care services among adults with learning disabilities: a scoping review. *Ann Stomatol (Roma)* 2017;7:52-59.65.

65. O’Malley L, Macey R, Allen T, Brocklehurst P, Thomson F, Rigby J, Lalloo R, Tomblin Murphy G, Birch S, Tickle M. Workforce Planning Models for Oral Health Care: A Scoping Review. *JDR Clin Trans Res* 2022;7:16-24.

66. Poudel P, Griffiths R, Wong VW, Arora A, George A. Knowledge and practices of diabetes care providers in oral health care and their potential role in oral health promotion: A scoping review . *Diabetes Res Clin Pract* 2017;130:266-277.

67. Saadatfar N, Jadidfard MP. An overview of the methodological aspects and policy implications of willingness-to-pay studies in oral health: a scoping review of existing literature. *BMC Oral Health* 2020;20:323.

68. Sanghavi A, Siddiqui NJ. Advancing oral health policy and advocacy to prevent childhood obesity and reduce children's consumption of sugar-sweetened beverages. *J Public Health Dent* 2017;77:S88-S95.

69. Schaefer R, Barbiani R, Nora CRD, Viegas K, Leal SMC, Lora PS, Ciconet R, Micheletti VD. Adolescent and youth health policies in the Portuguese-Brazilian context: specificities and approximations. *Cien Saude Colet* 2018;23:2849-2858.

70. Sekulic S, Theis-Mahon N, Rener-Sitar K. A systematic scoping review of oral health models. *Qual Life Res* 2019;28:2651-2668.

71. Seyedzadeh Sabounchi S, Seyedzadeh Sabounchi S, Cosler LE, Atav AS. Opioid prescribing and misuse among dental patients in the US: a literature-based review. *Quintessence Int* 2020;51:64-76.

72. Shrivastava R, Couturier Y, Girard F, Papineau L, Emami E. Two-eyed seeing of the integration of oral health in primary health care in Indigenous populations: a scoping review. *Int J Equity Health* 2020;19:107.

73. Shrivastava R, Power F, Tanwir F, Feine J, Emami E. University-based initiatives towards better access to oral health care for rural and remote populations: A scoping review. *PLoS One* 2019;14:e0217658.

74. Singh A, Harford J, Schuch HS, Watt RG, Peres MA. Theoretical basis and explanation for the relationship between area-level social inequalities and population oral health outcomes - A scoping review. *SSM Popul Health* 2016;2:451-462.

75. Tella AJ, Olanloye OM, Ibiyemi O. Potential of teledentistry in the delivery of oral health services in developing countries. *Ann Ib Postgrad Med* 2019;17:115-123.

76. Ummer-Christian R, Iacono T, Grills N, Pradhan A, Hughes N, Gussy M. Access to dental services for children with intellectual and developmental disabilities - A scoping review. *Res Dev Disabil* 2018;74:1-13.

77. VanMalsen JR, Figueiredo R, Rabie H, Compton SM. Factors Associated with Emergency Department Use for Non-traumatic Dental Problems: Scoping Review. *J Can Dent Assoc* 2019;84:j3.

78. Villarosa AC, Villarosa AR, Salamonson Y, Ramjan LM, Sousa MS, Srinivas R, Jones N, George A. The role of indigenous health workers in promoting oral health during pregnancy: a scoping review. *BMC Public Health* 2018;18(1):381.

79. Villarosa AR, George D, Ramjan LM, Srinivas R, George A. The role of dental practitioners in addressing overweight and obesity among children: A scoping review of current interventions and strategies. *Obes Res Clin Pract* 2018;12:405-415.

80. Weijs C, Lang R, Lorenzetti DL, Milaney K, Figueiredo R, Smith LB, McLaren LB. The Relation Between Exposure to Intimate Partner Violence and Childhood Dental Decay: A Scoping Review to Identify Novel Public Health Approaches to Early Intervention. *J Can Dent Assoc* 2019;84:j5.

81. Worsley D, Robinson PG, Marshman Z. Access to urgent dental care: A scoping review. *Community Dent Health* 2017;34:19-26.

**List of excluded studies**

1. Ramírez-De Los Santos S, López-Pulido EI, Medrano-González IC, Becerra-Ruiz JS, Alonso-Sanchez CC, Vázquez-Jiménez SI, Guerrero-Velázquez C, Guzmán-Flores JM. Alteration of cytokines in saliva of children with caries and obesity. *Odontology* 2021;109:11-17.
2. de Almeida CVVB, Pintado-Palomino K, Fortes JHP, da Motta RJG, de Freitas BN, Matsumoto W, de Oliveira Cavalcanti MTM, Alves J, Tirapelli C. Digital photography vs. clinical assessment of resin composite restorations. *Odontology* 2021;109:184-192.
3. Solow R. Systematic review versus structured critical analysis. *Cranio* 2021;39:189-201.
4. Makansi N, Blaizot A, Vergnes JN, Morris M, Rousseau J, Bedos C. A scoping review on dental clinic accessibility for people using wheelchairs. *Spec Care Dentist*

2021;41:329-339.
5. Cano-Ibáñez N, Zolfaghari Y, Amezcua-Prieto C, Khan KS. Physician-Patient Language Discordance and Poor Health Outcomes: A Systematic Scoping Review. *Front Public Health* 2021;9:629041.
6. Sabharwal A, Stellrecht E, Scannapieco FA. Associations between dental caries and systemic diseases: a scoping review. *BMC Oral Health* 2021;21(1):472.

7. Leadbeatter D, Holden ACL. How are the social determinants of health being taught in dental education? *J Dent Educ* 2021;85:539-554.
8. García-Pola M, Pons-Fuster E, Suárez-Fernández C, Seoane-Romero J, Romero-Méndez A, López-Jornet P. Role of Artificial Intelligence in the Early Diagnosis of Oral Cancer. A Scoping Review. *Cancers (Basel)* 2021;13:4600.
9. Bradbury-Jones C, Isham L, Morris AJ, Taylor J. The "Neglected" Relationship Between Child Maltreatment and Oral Health? An International Scoping Review of Research. *Trauma Violence Abuse* 2021;22:265-276.
10. Farrokhi F, Mohebbi SZ, Farrokhi F, Khami MR. Impact of COVID-19 on dental education- a scoping review. *BMC Med Educ* 2021;21(1):587.
11. Bhaumik D, Manikandan D, Foxman B. Cariogenic and oral health taxa in the oral cavity among children and adults: A scoping review. *Arch Oral Biol* 2021;129:105204.
12. Adeoye J, Tan JY, Ip CM, Choi SW, Thomson P. "Fact or fiction?": Oral cavity cancer in nonsmoking, nonalcohol drinking patients as a distinct entity-Scoping review. *Head Neck* 2021;43:3662-3680.
13. Chavis SE, Wu E, Munz SM. Considerations for Protective Stabilization in Community General Dental Practice for Adult Patients with Special Healthcare Needs. *Compend Contin Educ Dent* 2021;42:134-138.
14. Lieffers JRL, Vanzan AGT, Rover de Mello J, Cammer A. Nutrition Care Practices of Dietitians and Oral Health Professionals for Oral Health Conditions: A Scoping Review. *Nutrients* 2021;13:3588.

15. Guerrero EG, Kaplan CD, Gruß I, Frantsve-Hawley J, Fellows JL, Yosuf N, Polk DE. Applying a scoping review approach for identifying effective implementation strategies in oral health settings. *J Clin Transl Sci* 2021;5:e187.
16. Sanglard LF, Oliveira LB, Massignan C, Polmann H, De Luca Canto G. Evaluating pain, fear, anxiety or stress/distress using children's drawings in paediatric dentistry: a scoping review. *Eur Arch Paediatr Dent* 2022;23:199-222.
17. Dawett B, Deery C, Banerjee A, Papaioannou D, Marshman, Z. A scoping literature review on minimum intervention dentistry for children with dental caries. *Br Dent J* 2022 [Online ahead of print.]

18. Chandel T, Alulaiyan M, Farraj M, Riedy CA, Barrow JR, Brennan L, Thompson L, Bass MB, Chamut S. Training and educational programs that support geriatric dental care in rural settings: A scoping review. *J Dent Educ* 2022 [Online ahead of print.]

19. O’Malley L, Macey R, Allen T, Brocklehurst P, Thomson F, Rigby J, Lalloo R, Tomblin Murphy G, Birch S, Tickle M. Workforce Planning Models for Oral Health Care: A Scoping Review. *JDR Clin Trans Res* 2022;7:16-24.

20. Kshirsagar MM, Dodamani AS, Dodamani GA, Khobragade VR, Garg Y, Deokar RN. Teledentistry: A new horizon in COVID-19 pandemic for oral health. *Int J Clin Pediatr Dent* 2021;14:441-442.
